# The epidemiology of *Clostridium perfringens* type A on Ontario swine farms, with special reference to *cpb2*-positive isolates

**DOI:** 10.1186/1746-6148-8-156

**Published:** 2012-09-04

**Authors:** Gloria Chan, Abdolvahab Farzan, Glenn Soltes, Vivian M Nicholson, Yanlong Pei, Robert Friendship, John F Prescott

**Affiliations:** 1 Department of Population Medicine, University of Guelph, Guelph, ON, N1G 2W1, Canada; 2 Department of Pathobiology, University of Guelph, Guelph, ON, N1G 2W1, Canada

**Keywords:** *Clostridium perfringens* type A, *cpb2*, Pig, Epidemiology

## Abstract

**Background:**

There is poor understanding of most aspects of *Clostridium perfringens* type A as a possible cause of neonatal diarrhea in piglets, and the prevalence and types of *C. perfringens* present on Ontario swine farms is unknown. To study the prevalence of fecal *C. perfringens* and selected toxin genes, 48 Ontario swine farms were visited between August 2010 and May 2011, and 354 fecal samples were collected from suckling pigs, lactating sows, weanling pigs, grower-finisher pigs, and gestating sows, as well as from manure pits. The fecal samples were cultured quantitatively, and toxin genes were detected by real-time multiplex polymerase chain reaction (PCR).

**Results:**

In mixed multivariable linear analysis, log_10_*C. perfringens* in fecal samples from suckling pigs were higher than that of weanling pigs, grower-finisher pigs, and manure pit samples (*P <*0.05). In mixed multivariable logistic analysis, the *C. perfringens* isolates recovered from lactating sows (OR = 0.069, P <0.001), gestating sows (OR = 0.020, P <0.001), grower-finishers (OR = 0.017, P <0.001), and manure pits (OR = 0.11, P <0.001) were less likely to be positive for the consensus beta2 toxin gene *cpb2* compared to the isolates from suckling pigs. The prevalence of *cpb2* in the isolates recovered from weanlings did not differ significantly from suckling pigs. *C. perfringens* isolates that were positive for *cpb2* were more likely to carry the atypical *cpb2* gene (*atyp-cpb2*) (OR = 19, *P <*0.001) compared to isolates that were negative for *cpb2*. Multivariable analysis did not identify farm factors affecting the presence of consensus *cpb2* and *atyp-cpb2* genes.

**Conclusions:**

This study provides baseline data on the prevalence of *C. perfringens* and associated toxin genes in healthy pigs at different stages of production on Ontario swine farms. The study suggests that if *C. perfringens* type A are involved in neonatal enteritis, there may be strains with specific characteristics that cannot be identified by the existing genotyping system.

## Background

*Clostridium perfringens* type A is considered by some to be one of the most common causes of diarrhea in neonatal pigs [1,2]. However, the pathogenic basis of *C. perfringens* type A diarrhea is unclear, and the current diagnostic methods for this disease are not specific.

*Clostridium perfringens* are ubiquitous Gram-positive anaerobes that can be isolated from many environments, and their spore-forming ability allows them to persist in the swine ecosystem [2]. Currently, *C. perfringens* are divided into five toxinotypes (A to E) depending on their production of four major toxins. Isolates of any toxinotype may also produce enterotoxin (CPE) and beta2 toxin (CPB2) [3].

In the past decade, diagnosis of neonatal piglet diarrhea due to *C. perfringens* type A has increased, and has been associated with increased pre-weaning mortality [1]. Disease can develop in suckling pigs in the first week of life, and typical clinical signs include non-hemorrhagic, mucoid diarrhea seen within 48 h of birth, and lasting for 5 d [2]. The current method of diagnosis is based on isolation of large numbers of *C. perfringens* type A possessing the consensus CPB2 gene (*cpb2*) detected by polymerase chain reaction (PCR) from the feces or intestinal contents, and the exclusion of other known causes of neonatal diarrhea [3]. There is currently no commercial test available for detecting CPB2 in isolates [1,3]. Most porcine isolates carry and express *cpb2 *[4].

There is poor understanding of most aspects of *C. perfringens* type A as a possible cause of neonatal diarrhea in piglets, and the prevalence and types of *C. perfringens* present on Ontario swine farms is unknown. The objectives of this study were to compare the prevalence of *C. perfringens* and selected toxin genes among pigs at different stages of production, and to identify farm factors affecting this prevalence.

## Methods

### Farm selection

Forty-eight farms in Ontario were conveniently selected and visited once or twice between two periods of sample collection from August to December 2010, and from January 2011 and May 2011. For the first period of sample collections, 28 farms were visited. One farm was visited twice in the first period, and visited again in the second period. In the second period, a total of 11 farms from the first period were visited again, and 20 farms were visited for the first time. Some farms that were visited from the first period were not re-visited because they did not have suckling pigs or they were no longer in business.

### Sample collection

In both periods of sample collection, for farrowing operations, pooled-fecal samples were collected from gestating sows, weanling pigs, and grower-finisher pigs. In addition, a pooled sample was taken from the manure pit. In the second period of sample collection, fecal samples were also collected from lactating sows and their litters. A lactating sow fecal sample was collected by randomly selecting a sow, and manually obtaining a rectal sample with a gloved hand. A pooled-fecal sample of the lactating sow’s litter was also obtained by selecting three suckling pigs in the litter and collecting the feces from the piglet’s rectum. If suckling pigs did not defecate sufficient material during the sampling process, fresh piglet feces were obtained from the farrowing area of the litter. Three fecal samples were collected from lactating sows, and three pooled fecal samples were collected from their respective litters from each farrowing operation.

A pooled-fecal sample of gestating sows was obtained by selecting 6 sows from different areas of the barn, and obtaining a rectal sample. A pooled-fecal sample of weanling pigs or grower-finisher pigs was obtained from randomly selecting 6 pens, observing the pigs defecating, and then immediately collecting the fresh feces from the floor of the pens. On farrow-to-feeder farms sampling was similar to farrow-to-finish farms except two pooled samples were collected from weaning pigs. On grower-finisher farms, 3 pooled-fecal samples were taken from the pigs present, as well as a pooled manure pit sample. All farms on the study used a liquid manure system and a sample from the holding tank or manure pit was obtained by filling a plastic bottle from the manure pit. Liquid manure from 3 locations in the pit and at 3 different depths was combined to create the pooled sample.

### Questionnaire

A survey was administered to collect information on each farm for different management factors including the following: type of operation (farrowing-to-finish, farrowing-to-feeder, or grower-finisher operation), total number of barns, herd size (number of sows, gilts, weanling pigs, and grower-finisher pigs), sow flow, weanling flow, grower-finisher flow (all-in/all-out or continuous), number of farrowing rooms and crates, the presence of other agricultural species (other species), drug usage and vaccination, history of diarrhea outbreak in suckling pigs or post-weaning pigs in the past year, and treatment of the diarrhea problem. A total of 46 farms were surveyed; two farms were not surveyed because they were no longer in business.

### Bacterial isolation

For bacterial culture, samples were weighed and 4–5 serial 10-fold dilutions were performed in phosphate buffered saline, pH 7.2. Fecal dilutions were plated onto selective SFP (Shahadi Ferguson Perfringens) medium (Difco, Detroit, MI, USA). The SFP media contains 5% egg yolk emulsion (Oxoid, Nepean, ON), 12 μg/ml kanamycin sulphate, and 30 IU/ml polymyxin B sulphate (SFP Selective Supplement, Oxoid). The plates were transferred to an anaerobic jar, with anaerobic atmosphere provided by GasPak (BD, Sparks, MD, USA) and incubated at 37°C overnight. Colonies with characteristic *C. perfringens* colonial and microscopic morphologies, as well as lecithinase activity on the egg yolk agar, were counted and counts converted to colony-forming units (CFU) per gram.

### Genotyping of isolates

*Clostridium perfringens* colony lysates were examined by multiplex polymerase chain reaction (PCR) for toxin genes using the method described by Albini and colleagues [5]. Five isolates per sample were genotyped for *cpa*, consensus *cpb2* (*cpb2*) and atypical *cpb2* (*atyp-cbp2)*. The isolates recovered during the first period of the study were also genotyped for the major toxin genes beta toxin (*cpb*), enterotoxin (*cpe*), epsilon toxin (*etx*), and iota toxin (*itx*), NetB toxin (*netB*) and large clostridial cytotoxin (*tpeL*). Since these were all found to be absent they were not examined in the second period. The primer sets for the major toxin genes and *cpb2* used were designed using the reported nucleotide sequences [5]. The primer sets for *netB*, *tpeL*, and *atyp-cpb2* were designed for this study using standard approaches and appropriate bacterial control strains. The Roche LightCycler® 480 SW 1.5 software package was used to analyze the data. Relevant positive control strains were included in all batched PCR tests.

### Data analysis

The data were entered in a spreadsheet (Microsoft Excel 2007; Microsoft Corp., Redmond, WA) and imported to Stata 10 Intercooled for Windows XP (StataCorp LP, College Station, TX) for statistical analysis.

Three outcomes were measured, which included *C. perfringens* type A (*Cp*A) count in fecal samples, the presence of *cpb2*-carrying *C. perfringens* type A (*cpb2*-*Cp*A) isolates in fecal samples, and the presence of *atyp-cpb2-*carrying *C. perfringens* type A (atyp-cpb2-*Cp*A) isolates in fecal samples. The independent variables included in multivariable analysis were stage of production (main effect), type of operation, total number of barns, herd size (small or <1000, medium or ≥ 1000–3000, and large or >3000), and the presence of other species.

A mixed linear regression modeling method with farm as a random effect was used to analyze the association between *CpA* count in fecal samples and independent variables. Intra-class correlation coefficient was calculated as $\text{ICC}={\sigma^{2}}_{\text{farm}}/\left({\sigma^{2}}_{\text{farm}}+{\sigma^{2}}_{\text{sample}}\right)$. In addition, two separate logistic regression models were fitted to investigate the association between the presence of *cpb2*-*Cp*A and *atyp*-*cpb2*-*Cp*A in fecal samples and independent variables. Intra-cluster coefficient was calculated as $\text{ICC}={\sigma^{2}}_{\text{farm}}/\left({\sigma^{2}}_{\text{farm}}+\pi^{2}/3\right)$ by assuming that sample level variance on the logit scale was π^2^/3, π = 3.1416.

Univariable analysis of independent variables and their association with *Cp*A count, and presence of *cpb2*-*Cp*A and *atyp-cpb2*-*Cp*A isolates in fecal samples was performed using a single linear or logistic regression. Variables with P <0.20 were selected for inclusion in multivariable analyses which was modeled at the sample level with farm as random variable.

A manual stepwise procedure was used to build the multivariable models. Pair-wise correlation coefficients were calculated between independent variables, and coefficients with an absolute value greater than 0.8 were considered colinear. If two independent variables were significantly correlated, the more informative variable was used in the final model. A variable was identified as a confounder if it changed the coefficient of the main effects by 20% or more when the potential confounder variable was removed. If a variable was determined to be a confounder, it was included in the final model regardless of its statistical significance. Interaction was evaluated between all independent variables and the main effects model. Each interaction term was assessed for statistical significance with the main effects, and the interaction terms with *P <*0.05 were included in the final model. Interaction terms that were not significant in the final model were removed if removal of the interaction term did not result in a significant change in the likelihood ratio test (logistic regression) and partial F-test (linear regression).

For linear multivariable analysis, a log_10_ transformation was used on the *C. perfringens* count (CFU/g) in order to meet the normal distribution and homoscedasticity assumptions for the outcome of interest. The Cook-Weisberg test was performed for the linear multivariable model with farm as a fixed effect, and the assumption of homoscedasticity was met if *P >*0.05. The Shapiro-Wilk normality test was used to test the normal distribution of log_10_ CFU/g with farm as a fixed effect, and the assumption of normality was met if *P >*0.05. The standardized residuals were graphically assessed for outliers, and the leverage values were graphically assessed.

The logistic regression multivariable model with farm as a fixed effect for the presence of *cpb2* in *C. perfringens* type A isolates was assessed for goodness-of-fit, which was indicated by *P >*0.05 in the Hosmer-Lemeshow test. Pearson residuals were graphically assessed for outliers, and the leverage values were graphically assessed.

The Spearman’s rank correlation test was used to analyze the association between the toxin genes in lactating sows and their litters.

## Results

A total of 354 fecal samples were collected, distributed between the sources shown in Table 1. Overall, *Clostridium perfringens* was isolated from 225 (64%) of 354 fecal samples (98% of suckling piglets, 34% of weanling pigs, 18% of grower-finisher pigs, 89% of gestating sows, 96% of lactating sows, and 75% of manure pit samples) (Table 1). A farm was considered positive for *C. perfringens* if *cpa* was detected in the isolate of at least one fecal sample, and a farm was considered positive for consensus *cpb2* if it was detected in the *C. perfringens* isolate of at least one fecal sample. A total of 42 farms (87.5%) were positive for *C. perfringens*, and 25 farms (52%) were positive for *cpb2*. *C. perfringens* was not recovered from samples collected on 6 (12.5%) of 48 farms, all of which were grower-finisher operations. Within-herd prevalence of *C. perfring*ens type A ranged between 0 and 100% with a mean of 53.5 ± 32%.

**Table 1:** **Log**_**10**_**CFU/g of*C. perfringens*in fecal samples and the positive proportion of toxin genes**

	**Log**_**10**_**(CFU/g)**	**Percentage of positive samples**		**Total No. of Samples**
	**Mean ± SD**	***Cpb2***	***Atyp-cpb2***	
Suckling pigs	5.0 ± 1.70	77	44	57
Lactating Sows	4.0 ± 1.86	26	16	57
Gestating Sows	4.3 ± 1.85	5	0	37
Weanling pigs	1.3 ± 1.87	18	7	44
Grower-finisher pigs	0.6 ± 1.24	2	5	99
Manure Pit	2.7 ± 1.82	27	17	60
Total	2.7 ± 2.38	25	15	354

### *Clostridium perfringens* count (log_10_ CFU/g)

The mean of log_10_ CFU/g of *Clostridium perfringens* in fecal samples is shown in Table 1. The total mean count was 2.7 ± 2.4 log_10_ CFU/g. The distribution of independent variables on 46 surveyed farms is shown in Table 2. Independent variables with *P <*0.20 that were initially included in the multivariable linear regression analysis were the stage of production, type of operation, total herd size, presence of other species, and sampling period. The type of operation and total herd size did not significantly affect the *C. perfringens* count of fecal samples in the final model (*P >*0.05). The log_10_*C. perfringens* count for fecal samples collected from the first and second sampling period was not significantly different (*P >*0.05); the sampling period was included in the final model because *C. perfringens* count in the samples collected from different stage of production was confounded by sampling period. In multivariable analysis, the log_10_*C. perfringens* count was higher in suckling pig fecal samples compared to that of weanling pigs, grower-finisher pigs, and the manure pit (*P* <0.05) (Table 3). The log_10_*C. perfringens* count was higher in gestating sow samples, lactating sow samples, or the manure pits, respectively, compared to that of weanling pigs and grower-finisher pigs (*P* <0.05). In the final model, *C. perfringens* count in fecal samples collected from sows (both gestating and lactating) was interacted by the presence of other species on farms (*P <*0.05) (Table 3). Overall, the log_10_*C. perfringens* count was higher in the fecal samples collected from sows on farms where other species were absent. The log_10_*C. perfringens* in gestating sows versus piglets was 4.1 = [(−1.35) + (−0.93) + 1.5+ 4.9 (constant)] and 5.0 = [(−1.35) + 1.5+ 4.9 (constant)] in the presence and absence of other species, respectively. Similarly, the log_10_*C. perfringens* in lactating sows versus piglets was 3.88 = [(−1.93) + (−0.93) + 1.84+ 4.9 (constant)] and 4.81 = [(−1.93) + 1.84+ 4.9(constant)] in the presence and absence of other species, respectively.

**Table 2:** The distribution of independent variables among 46 surveyed farms

**Independent Variable**		**Percentage of C*pb2*-positive farms**	**Univariable analysis*P*-value (sample level)**
Type of operation (sample n = 220)	Farrowing (farm n = 30)	73	0.012
	Grower-finisher (farm n = 16)	25	
Other species (sample n = 212)	Not present (farm n = 23)	57	0.004
	Present (farm n = 23)	57	
Herd Size (sample n = 217)	Small (<1000) (farm n = 9)	33	Referent
	Medium (≥1000-3000) (farm n = 28)	61	0.272
	Large (>3000) (farm n = 9)	67	0.018
Sow Flow (sample n = 84)	All-in/all-out (farm n = 16)	75	0.417
	Continuous (farm n = 14)	71	
Weanling Flow (sample n = 15)	All-in/all-out (farm n = 23)	70	0.326
	Continuous (farm n = 8)	87.5	
Grower-Finisher Flow (sample n = 20)	All-in/all-out (farm n = 27)	48	0.327
	Continuous (farm n = 13)	54	
Antibiotic usage (sample n = 66)	Yes (farm n = 7)	100	0.304*
	No (farm n = 23)	65	
Diarrhea (sample n = 58)	Yes (farm n = 11)	82	0.792*
	No (farm n = 19)	68	

*Exact logistic regression.

**Table 3:** **Multivariable linear regression model of factors associated with fecal*C. perfringens*count (log**_**10**_**CFU/g)**

**Variables**		**Coefficient**	**Standard Error**	**95% Confidence Interval**	***P*-value**
Stage	Suckling pig	Referent			
	Lactating sow	−1.9	0.367	(−2.7)-(−1.2)	<0.001
	Gestating sow	−1.4	0.438	(−2.2)-(−0.50)	0.002
	Weanling	−3.8	0.398	(−4.6)-(−3.1)	<0.001
	Grower-finisher	−4.4	0.413	(−5.2)-(−3.6)	<0.001
	Manure pit	−1.7	0.403	(−2.5)-(−0.93)	<0.001
Other species	Absent	Referent			
	Present	−0.93	0.484	(−1.9)-(−0.019)	0.055
	Present x Gestating sow	1.5	0.626	0.30-2.75	0.015
	Present x Lactating sow	1.8	0.533	0.79-2.9	0.001
Sampling Period	First	Referent			
	Second	0.34	0.234	(−0.12)-(−0.80)	0.144

The Intraclass Correlation Coefficient (ICC) was 0.24, indicating a low level of clustering in which the within-farm variation of the *C. perfringens* count accounted for almost a quarter of the total variation of the count.

Potential outliers were identified for two farms, of which the weanling fecal samples had a *C. perfringens* count of 6.0 and 6.7 log_10_ CFU/g, respectively. Potential extreme observations were identified for two farms, of which *C. perfringens* was not isolated from weanling fecal samples.

### *Clostridium perfringens*-associated toxin genes

The distribution of *cpb2* and *atyp-cbp2* genes among *Clostridium perfringens* isolates cultured from fecal samples is presented in Table 1.

The independent variables initially included in the multivariable logistic analysis were the stage of production, presence of *atyp-cpb2*, type of operation, presence of other species, total herd size, and sampling period. The type of operation, presence of other species, and total herd size did not significantly affect the likelihood of detecting *cpb2*-positive *C. perfringens* isolates in the final model (*P >*0.05). The variable of vaccination was not included in the statistical analysis because none of the farms surveyed vaccinated sows or pigs for *C. perfringens*.

*Cpb2* was more likely found in the isolates of suckling pigs compared to those of lactating sows, gestating sows, and grower-finisher pigs, and manure pits (*P* <0.05) (Table 4). The likelihood of finding *cpb2* in *C. perfringens* isolates recovered from weanling pigs was higher compared to that of gestating sows (OR = 0.077, *P =* 0.011) and grower-finisher pigs (OR = 0.065, *P* = 0.027).

**Table 4:** **Multivariable logistic regression model of factors associated with*cpb2*-positive*C. perfringens*isolates**

**Variables**		**Odds Ratio**	**Standard Error**	**95% Confidence Interval**	***P*-value**
Stage	Suckling pig	Referent			
	Lactating sow	0.069	0.0437	0.020-0.24	<0.001
	Gestating sow	0.020	0.0189	3.2 × 10^-3^-0.13	<0.001
	Weanling	0.26	0.216	0.052-1.3	0.103
	Grower-finisher	0.017	0.0185	2.0 × 10^-3^-0.14	<0.001
	Manure pit	0.11	0.0677	3.2 × 10^-2^-0.37	<0.001
*Atyp-cpb2*	Negative	Referent			
	Positive	19	12.0	5.7-65	<0.001

The Intraclass Correlation Coefficient (ICC) that compared the within-farm and between-farm variance was 0.24 and 0.25, respectively for linear regression and logistic regression, indicating a low level of clustering in which the within-farm variation of *C. perfringens* count and the presence of *cpb2* accounted for almost a quarter of the total variation.

The likelihood of detecting *atyp-cpb2* was higher in *cpb2*-positive isolates compared to *cpb2*-negative isolates (*P <*0.001) (Table 4). The percentages of samples that had *atyp-cpb2* were 51% in *cpb2*-positive samples, and 6% in *cpb2*-negative samples.

*Clostridium perfringens* isolates recovered from lactating sows (OR = 0.20, P = 0.001), weanlings (OR = 0.20, P = 0.039), and the manure pits (OR = 0.32, P = 0.022) were less likely to be positive for *atyp-cpb2* than isolates recovered from suckling pigs. The correlation of *cpb2* or *atyp-cpb2* in isolates of lactating sows and in isolates from their litters was not statistically significant.

Potential outliers were identified for two farms. On one farm, *cpb2* nor *atyp-cpb2* were detected in the *C. perfringens* isolates of a suckling pig fecal sample. On the other farm, *cpb2* was not detected in the *atyp-cpb2* positive *C. perfringens* isolates of the manure pit fecal sample. Potential extreme observations were identified for one farm, of which the *cpb2-*positive *C. perfringens* isolates were only recovered from two of three suckling pig fecal samples.

The isolates recovered during the first period of the study were all negative for *cpb*, *cpe*, *etx*, *itx*, *netB* and *tpeL*. For this reason, they were not examined in the second period.

## Discussion

The current study of enteric *Clostridium perfringens* in pigs identified novel findings that may increase the understanding of the epidemiology of infection and possible diagnosis of the suckling pig diarrhea thought to be caused by this organism. *Clostridium perfringens* type A is speculated to be an emerging pathogen associated with neonatal piglet diarrhea [6-9], although not all prevalence studies conducted on infectious causes of piglet diarrhea have included *C. perfringens* type A in the analysis [6,10,11]. Isolates recovered from diarrheic piglets could not be differentiated genetically from isolates recovered from non-diarrheic piglets in the United States [12] or on genotype in Canada [13].

This study is the first to examine systematically the quantitative prevalence of *C. perfringens,* as well as an expanded range of toxin genes, in pigs of different ages on multiple farms. Our results showed that the number of *C. perfringens* type A isolates was higher in suckling pigs compared to pigs in other stages of production. High numbers of *C. perfringens* were also isolated from gestating and lactating sows. The pattern of decline in colonization by *C. perfringens* with age has been noted previously in the intestine of individual pigs but not at the farm level [14]. The decrease in *C. perfringens* count for fecal samples in weanling and grower-finisher pigs was generally expected, since the population of *C. perfringens* decreases in the pig intestine as other bacterial species establish their populations [2]. Host-related factors that affect the decline in colonization may include diet, changes in large intestinal microflora with time, age-related changes in host physiology, or the development of intestinal immunity. The decline in numbers of *C. perfringens* in pigs in the grower-finisher stage may also be partly due to changes in management factors including the use of antimicrobials during the nursery and grower-finisher stages. The effect of other species on the farm in increasing *C. perfringens* counts in sows (Table 3) was an unexpected observation that factors other than “host” can affect colonization by this organism.

Many *C. perfringen*s isolated from pigs are relatively unusual among isolates from different animal sources in that they belong to a clonal population [15]. Unusually, these isolates are characterized both by being consensus *cpb2* positive and by expressing CPB2 [4]. There thus appears to be an adaptation of this lineage to swine. Although we did not however examine the isolates made here for their phylogenetic relationships, on the basis of the present study, the adaptation of this lineage appears to be to the young pig, and notably to the suckling piglet. This relationship of *cpb2*-positive *C. perfringens* to the young piglet (Tables 2, 4) has not previously been demonstrated at the farm level. Most of the suckling pig isolates in this study were positive for consensus *cpb2* as noted in other studies [4,7-9]. The gestating sow isolates were usually not positive for *cpb2* and lacked the *atyp-cpb2* gene that was found in lactating sow and suckling pig isolates, which appear to be specifically colonized by *cpb2*-positive strains [3]. It is possible that the sows in the lactating stage acquired *cpb2*-positive *C. perfringens* strains from suckling pigs in the litter, but that strains were not always maintained when the suckling pigs were weaned and sows returned to the gestating stage. However, there was no significant correlation of the presence of *cpb2* and *atyp-cpb2* in *C. perfringens* type A isolates from sows and their litters, suggesting that the source of the strains in suckling pigs may be different from sows, or that *cpb2* and/or *atyp-cpb2* positive isolates are more likely to proliferate in suckling pigs. Interestingly, there was a dominance of *atyp-cpb2* isolates in *C. perfringens* isolated from grower-finisher pigs. It is possible that other factors contributed to the decline in consensus *cpb2*-positive *C. perfringens* isolates seen in grower-finisher pigs, such as antimicrobial usage; a negative association between *cpb2*-positive *C. perfringens* isolates and resistance to erythromycin and clindamycin was documented in an Ontario study [16]. The isolates from the manure pit were unexpectedly similar to isolates from suckling pigs, reasons for which are unclear.

This study identified genes in the most abundant isolate of *C. perfringens*, so that the less abundant genotypes may not have been detected. *C. perfringens* isolates positive for *cpb2* were more likely to be positive for *atyp-cpb2*, suggesting a linkage between these genes. Further research on the genetic diversity of *C. perfringens* type A isolates in clinically healthy and diarrheic suckling pigs, and the conditions of which *cpb2* and *atyp-cpb2* toxin genes are expressed, may help clarify the role of this agent in neonatal piglet diarrhea.

Since the farms in our study were conveniently selected, the results may not apply to the general population of Ontario swine farms. The difference in bacterial count of *C. perfringens* in fecal samples from pigs at different stages of production might have been affected by the sampling procedure, since samples for weanling pigs and grower-finisher pigs were collected from feces on the pen floor whereas the samples for sows and suckling pigs were collected from the rectum. The fecal samples collected from gestating sows, weanling pigs, grower-finisher pigs, and the manure pits were pooled, so it is conceivable that some samples were misclassified. Fecal samples collected from farrowing operations in the first period of the study did not include those of suckling pigs or lactating sows, which could affect the farm-level sensitivity for the detection of *cpb2*-positive *C. perfringens* isolates from these farms.

This is the first time that *C. perfringens* type A from swine has been examined for *netB* and *tpeL*, genes that are common in isolates from chickens with necrotic enteritis [17]. Their absence further supports the idea of host adaptation of strains of *C. perfringens*, as apparent here in the association of *cpb*2-positive strains with piglets. The absence of *cpb* is consistent with the absence of *C. perfringens* type C in enteric disease in swine and other animals in Ontario. The lack of *cpe* in *C. perfringens* isolates confirms earlier studies [18,19].

## Conclusions

To our best knowledge, this is the first study on the epidemiology of *C. perfringens* in the swine population. This study provides baseline data from healthy pigs at different stages of production as well as in the manure pits for future studies to investigate the role of *C. perfringens* in swine enteric diseases. The study suggests that if C*. perfringens* type A are involved in neonatal enteritis, there may be strains with specific characteristics that cannot be identified by the existing genotyping system. Further work is required to determine whether this is the case.

## Competing interests

The authors declare that they have no competing interests.

## Authors’ contributions

JP, RF, and AF designed the study. GC and AF performed the data collection, and statistical analysis. GS, VN, and YP performed the bacterial isolation. GS and VN performed the multiplex polymerase chain reaction. All authors contributed to drafting and revising of the manuscript. All authors read and approved the final manuscript.
